# Comparison of depressive symptoms among healthcare workers in high-risk versus low-risk areas during the first month of the COVID-19 pandemic in China

**DOI:** 10.3389/fpsyt.2023.1154930

**Published:** 2023-06-13

**Authors:** Emma Yun Zhi Huang, Lillian Liang-Chi Li, Aderonke Odetayo, Xing-Wei Zhang, Jonathan Ka Ming Ho, Shun Chan, Vivian Ching Man Pang, Lorna Kwai Ping Suen, Simon Ching Lam

**Affiliations:** ^1^Nutrilite Health Institute, Shanghai, China; ^2^Department of Rehabilitation Science, Hong Kong Polytechnic University, Hung Hom, Hong Kong SAR, China; ^3^School of Nursing, Tung Wah College, Kowloon, Hong Kong SAR, China; ^4^Zhongshan Polytechnic, Zhongshan, China; ^5^School of Nursing and Health Studies, Hong Kong Metropolitan University, Ho Man Tin, Hong Kong SAR, China

**Keywords:** COVID-19, health personnel, depression, personal protective equipment, health belief model

## Abstract

**Introduction:**

The psychological health of healthcare workers (HCWs) has become a significant concern, particularly during the initial stage of a pandemic. This study compared the depressive symptoms among HCWs in high-risk areas (HRAs) and low-risk areas (LRAs) with matching demographics.

**Methods:**

A cross-sectional study was employed to compare the depressive symptoms (Patient Health Questionnaire score ≥ 10), workplace environment characteristics, the Health Belief Model (HBM) and socio-demographics of the HCWs working in HRAs and LRAs in several accessible regions (mainly Hubei Province and Guangdong–Hong Kong–Macao Greater–Bay–Area) in China. Eight hundred eighty-five HCWs were recruited for unmatched analysis between March 6 and April 2, 2020. After matching with occupation and years of service using a 1:2 ratio, 146 HCWs in HRAs and 290 HCWs in LRAs were selected for matched analysis. Subgroup analyzes were performed using two individual logistic regressions to delineate the associated factors in LRAs and HRAs, respectively.

**Results:**

HCWs in LRAs (Prevalence = 23.7%) had 1.96 times higher odds of depressive symptoms than those in HRAs (Prevalence = 15.1%) after adjusting for occupation and years of service (*p* < 0.001). Significant differences in workplace environment characteristics (*p* < 0.001) and the 5-dimension of the HBM of HCWs (*p* < 0.001 to *p* = 0.025) were found between HRAs and LRAs.

Logistic regression showed that workers with years of service between 10 and 20  years (OR:6.27), ever had contact with COVID-19 patients (OR:14.33) and had higher scores of “perceived barrier” of HBM (OR:4.48) predicted depressive symptoms in HRAs while working in pneumology departments and infectious disease units (OR:0.06), and high “self-efficacy” in the HBM (OR:0.13) was a protective factor against depressive symptoms.

Contrarily, in LRAs, those HCWs who worked in ICUs (OR:2.59), had higher scores of “perceived susceptibility toward the COVID-19 outbreak” (OR:1.41), “perceived severity of the pandemic” (OR:1.25), and “perceived barriers of wearing masks” (OR:1.43) in the HBM predicted depressive symptoms. High “cues to action” (OR:0.79), and better “knowledge” (OR:0.79) in the HBM were protective factors against depressive symptoms.

**Conclusion:**

The risk of depressive symptoms of HCWS was double in LRAs than in HRAs in the first month of the COVID-19 pandemic. Furthermore, salient predictors for depressive symptoms among HCWs in HRAs and LRAs were very different.

## Introduction

The coronavirus disease (COVID-19) has become a global pandemic since December. 2019, resulting in massive loss of lives and posing an unprecedented challenge to global health (1, 2). Healthcare workers (HCWs) are at the core of this global fight against the COVID-19 pandemic. The enormous number of cases and fatalities brought on by this pandemic means that HCWs worldwide have been under work overload and mental stress (3), a situation which can lead to an increased prevalence of depressive symptoms among health professionals (4).

According to two latest systematic reviews conducted in Asia, the COVID-19 pandemic has posed a challenging problem among HCWs because of mental tiredness, burnout, dread, sadness, insomnia, and psychological stress, which may adversely affect both HCWs and patient safety (5, 6). The demanding work conditions coupled with the shortage of personal protective equipment and the fear of contracting the virus may enhance the risk of developing depressive symptoms among HCWs (7–9). Current local studies in China and international research on the depressive symptoms also indicated that HCWs were under higher psychological pressure (10–14), a finding which may be attributed to the high demand of work, a lack of readiness for such a pandemic, and the inadequate supply of occupational protective measures. Many studies compared depressive symptoms among HCWs who worked in different working environments [for example, high-risk area (HRAs) and low-risk areas (LRAs)] in Mainland China using convenience sampling as the data collection method (15). However, when this sampling method is used without matching the samples’ demographics, the analysis may be prone to error due to the presence of some essential confounders related to the workplace environment (15). This can lead to over–or under-estimation of the results.

Depressive symptoms among HCWs have been indicated to be closely related to years of work experience and type of occupation (16, 17). However, there are few comparative studies on the depressive symptoms among HCWs in relation to their risk of workplaces, such as in HRAs and LRAs. Owing to this comparative gap, determining the factors that affect HCWs’ depressive symptoms is difficult. Hence, this study aimed to compare the depressive symptoms among HCWs in HRAs and LRAs in China based on matched characteristics, and hence identify the associated factors that predict HCWs’ depressive symptoms specific to different workplaces.

## Methods

This comparative and cross-sectional study adopted a matching of socio-demographics approach to increase the rigor of comparison between HWCs in HRAs and LRAs. A null hypothesis was used that there is no difference of depressive symptoms between HWCs working in HRAs and LRAs.

### Participants

We conducted an online survey among HCWs working in HRAs and LRAs in China through various platforms (WenJuanXing, WeChat, and other Internet platforms) between March 6 and April 2, 2020, using the convenience sampling method. The World Health Organization officially declared “a Public Health Emergency of International Concern on January 30, 2020, and to characterize the outbreak as a pandemic on March 11, 2020” (18). Therefore, this study investigated the first month of the COVID-19 pandemic (18) on depressive symptoms and related situations among HCWs, where a pandemic is defined as an infectious disease spreading across several countries and affecting a higher-than-expected (usually very large) number of people.

### Settings

Since this was an online data collection method, the names of study places would be various (refer to the Supplementary Table A), namely Hubei Province and Guangdong–Hong Kong–Macao Greater Bay Area. An HRAs refers to the clinical environment where HCWs would routinely perform treatment or care for patients with confirmed or suspected COVID-19 cases, such as the infectious disease ward, intensive care unit, and accident and emergency department in a region with a known COVID-19 outbreak (19–21). An LRAs refers to the clinical environment wherein the HCWs were unlikely to or only occasionally have contacted some identified COVID-19 cases, such as the infirmary unit and rehabilitation ward (19–22). HCWs for regions that did not experience the COVID-19 outbreak were regarded as working for LRAs at the time of data collection (refer to the Supplementary Table B for details).

### Data collection

The survey link for the questionnaire was sent by our research team with invitation sentences to one or two doctors or nurses from the target hospital who, then distributed the questionnaire to their colleagues in other departments. The questionnaire comprised 35 items and can only be submitted after HCWs have completed all the questions. Each IP address was only allowed to submit the questionnaire once. Informed consent was obtained from the participants before starting the study. The inclusion criteria were as follows (1): HCWs who could understand the study purpose and agree to participate in this study on a voluntary basis, and (2) HCWs who routinely worked during the outbreak of COVID-19. A total of 885 questionnaires were collected from the study. Consequently, data of 146 and 739 HCWs were coded for HRAs and LRAs, respectively.

### Study tools

HCWs from HRAs and LRAs were surveyed using a Chinese self-reported questionnaire with five sections, i.e., socio-demographics, workplace environment characteristics, Patient Health Questionnaire (PHQ-9) (16, 23, 24), and the Health Belief Model (HBM) (25).

Socio-demographic variables such as gender, occupation, working department, education level, marital status, years of service (±1 year), contact with confirmed or suspected cases of COVID-19, and contact with patients infected with respiratory infectious diseases were also recorded for analysis.

Workplace environment characteristics were investigated with five items (1): the types of masks routinely used (2), the type of masks provided by the department (3), the types of masks that HCWs most wanted to wear (4), whether the protective equipment provided by the hospital is adequate, and (5) the satisfaction level of infection prevention training provided by the hospital. These items have been adopted and reported in a previous study conducted by Lam and his team (21).

The PHQ-9 was employed to assess the depressive symptoms of HCWs in HRAs and LRAs. The nine items comprising the PHQ-9 were measured with a four-point ordinal scale ranging from “0 = not at all” to “3 = nearly every day.” (23, 24). The total score could range from 0 to 27, and the severity of depression increases with the score (10–14 for moderate depression, 15–19 for moderately severe depression, and 20–27 for severe depression) (23). Results from a large population study in Hong Kong showed that PHQ-9 was effective in screening depressive symptoms (sensitivity 80%, specificity 92%) (23). In this study, the cutoff value of PHQ-9 ≥10 was defined as having a depressive symptom tendency, and < 10 was described as having no depressive symptom tendency (24). The reliability (Cronbach’s alpha = 0.89 for internal consistency) and validity (exploratory and confirmatory factor analysis used for construct validation) of PHQ-9 for the Chinese people were satisfactory (24).

The HBM (25) is a widely used social-psychological model which provides a valuable framework for investigating health behaviors and identifying essential health beliefs. This study adopted the questionnaire published by Bressington et al. (26) and Cheung et al. (27), their work being the first two studies that investigated the association between mental health and health beliefs globally and locally. The questionnaire consisted of 7 dimensions with 13 questions, including (i) perceived susceptibility toward the COVID-19 outbreak (3 items); (ii) perceived severity of the pandemic (2 items); (iii) perceived benefits of wearing masks (1 item); (iv) perceived barriers of wearing masks (2 items); (v) cues to action for self-protection (2 items); (iv) knowledge of the COVID-19 outbreaks (2 items); and (vii) self-efficacy of properly wearing a mask (1 item). Face, content validity, and construct validity (i.e., known-group method and exploratory factor analysis) was reported in previous studies with satisfactory results (26, 28).

### Statistical analysis

The data were inputted into an Excel spreadsheet, and SPSS statistical package version 28.0 software was used for data analysis. Study participants’ demographics and characteristics (categorical data) were analyzed using frequency and percentage. Continuous data were expressed as mean and standard deviation (SD). The differences in participants’ workplace environment characteristics, HBM scores, and PHQ-9 scores were analyzed using independent samples t-test and Chi-square test. For identifying the risk and exploring the associated factors of depressive symptoms among HCWs from HRAs and LRAs, multinomial logistic regression and binary logistic regression were applied. We defined the statistical significance as *p* < 0.05; all tests were two-sided.

## Results

### Socio-demographic characteristics

A total of 885 questionnaires were collected from this study. There were 146 HCWs in HRAs and 739 HCWs in LRAs. Table 1 presents the socio-demographic characteristics of both HRAs and LRAs groups before and after matching. According to the matching ratio of 1:2 for this cross-sectional study, 146 HCWs (37% male and 63% female) were grouped as the HRAs group. After matching with occupation and years of service, 290 HCWs (23.4% male and 76.6% female) were grouped as the LRAs group, for a total of 436 samples. The majority of HCWs were female (63.0–77.7%), licensed nurses (60.2–62.1%), married (64.3–76.9%), with bachelor’s degrees (60.9–65.8%), and with less than 20 years of service (82.5–93.8%; refer to Supplementary Table C for graphical illustration).

**Table 1:** Characteristics of the HCWs before and after matching.

	Unmatched		Matched^a^	
Characteristics	High-risk areas	Low-risk areas	High-risk areas	Low-risk areas
	(*N* = 146)	(*N* = 739)	*N* = 146	*N* = 290
Gender				
Male	54 (37.0%)	165 (22.3%)	54 (37.0%)	68 (23.4%)
Female	92 (63.0%)	574 (77.7%)	92 (63.0%)	222 (76.6%)
Occupation				
Doctor	52 (35.6%)	213 (28.8%)	52 (35.6%)	100 (34.5%)
Nurse	89 (61%)	445 (60.2%)	89 (61%)	180 (62.1%)
Other	5 (3.4%)	81 (11.0%)	5 (3.4%)	10 (3.4%)
Working department				
Internal medicine & surgery department	19 (13.0%)	196 (27.0%)	19 (13.0%)	65 (22.4%)
Other	29 (19.9%)	435 (60.0%)	29 (19.9%)	186 (64.1%)
ICU	67 (45.9%)	48 (6.6%)	67 (45.9%)	17 (5.9%)
Pneumology department and infectious disease	31 (21.2%)	46 (6.4%)	31 (21.2%)	22 (7.6%)
Education level				
Associate degree or below	32 (21.9%)	193 (26.2%)	32 (21.9%)	60 (20.7%)
Bachelor’s degree	96 (65.8%)	449 (60.9%)	96 (65.8%)	183 (63.1%)
Master’s degree or above	18 (12.3%)	95 (12.9%)	18 (12.3%)	47 (16.2%)
Marital status				
Unmarried/Other	46 (31.5%)	264 (35.7%)	46 (31.5%)	67 (23.1%)
Married	100 (68.5%)	475 (64.3%)	100 (68.5%)	223 (76.9%)
Year of service				
<10	67 (45.9%)	348 (47.1%)	67 (45.9%)	134 (46.2%)
10–20	69 (47.3%)	262 (35.4%)	69 (47.3%)	138 (47.6%)
>20	10 (6.8%)	129 (17.5%)	10 (6.8%)	18 (6.2%)
Ever contacted with COVID-19 patients				
No	34 (23.3%)	512 (69.3%)	34 (23.3%)	196 (67.6%)
Yes	112 (76.7%)	227 (30.7%)	112 (76.7%)	94 (32.4%)
Ever contacted with respiratory infectious diseases				
No	11 (7.5%)	147 (19.9%)	11 (7.5%)	53 (18.3%)
Not sure	5 (3.4%)	60 (8.1%)	5 (3.4%)	11 (3.8%)
Yes	130 (89.1%)	532 (72.0%)	130 (89.1%)	226 (77.9%)
Depressive symptoms				
No	124 (84.9%)	564 (76.3%)	124 (84.9%)	224 (77.2%)
Yes	22 (15.1%)	175 (23.7%)	22 (15.1%)	66 (22.8%)
Severity of depressive symptoms				
Non-depressive symptoms	124 (84.9%)	564 (76.3%)	124 (84.9%)	224 (77.2%)
Moderate depressive symptoms	15 (10.3%)	94 (12.7%)	15 (10.3%)	38 (13.1%)
Moderately severe depressive symptoms	6 (4.1%)	54 (7.3%)	6 (4.1%)	22 (7.6%)
Severe depressive symptoms	1 (0.7%)	27 (3.7%)	1 (0.7%)	6 (2.1%)

a In our study, occupation and year of service were matched.

Significant differences (i.e., gender, working department, ever had contacted with COVID-19 patients, and ever had contacted with respiratory infectious diseases) were consistently demonstrated in unmatched and matched samples for HCWs in HRAs and LRAs (*p* <  0.05; analysis not shown in Table 1). After matching, a cluster of variables, including occupation, education level, marital status, and years of service, revealed no significant difference between HRAs and LRAs groups.

### Comparison of depressive symptoms

The prevalence of depressive symptoms in matched HRAs and LRAs groups were 15.1 and 22.8%, respectively. In addition, a significant difference in depressive symptoms among HCWs in HRAs and LRAs was found only in unmatched samples *p* = 0.022) but not in matched samples (*p* = 0.054) in univariate analyzes.

As shown in Table 2, the risk of depressive symptoms in the unmatched sample was 1.93 times higher among the HCWs of LRAs relative to their HRAs counterparts [adjusted odds ratio (AOR): 1.93, 95%CI = 1.12–3.33]. A comparable and consistent result was also obtained in the matched sample (AOR: 1.96, 95%CI = 1.07–3.62). However, multinomial logistic regression indicated HCWs in two different workplaces did not predict the degree (i.e., moderate, moderately severe, and severe) of depressive symptoms in either unmatched or matched samples, adjusted or non-adjusted calculations.

**Table 2:** Results for logistic regression model using high-risk areas as a reference before and after matching.

	Depressive symptoms^a^		Moderate depressive symptoms^b^		Moderately severe depressive symptoms^b^		Severe depressive symptoms^b^	
	OR(95%CI)	AOR(95%CI)	OR(95%CI)	AOR(95%CI)	OR(95%CI)	AOR(95%CI)	OR(95%CI)	AOR(95%CI)
Unmatched								
Low-risk areas	1.75	1.93	1.38	1.54	1.98	2.31	5.94	4.69
	(1.08–2.84)	(1.12–3.33)	(0.77–2.46)	(0.80–2.95)	(0.83–4.70)	(0.89–5.96)	(0.80–44.10)	(0.58–38.07)
High-risk areas	1 [reference]	1 [reference]	1 [reference]	1 [reference]	1 [reference]	1 [reference]	1 [reference]	1 [reference]
Matched^c^								
Low-risk areas	1.66	1.96	1.40	2.04	2.03	1.72	3.32	2.30
	(0.98–2.82)	(1.07–3.62)	(0.74–2.65)	(0.98–4.23)	(0.80–5.14)	(0.60–4.95)	(0.40–27.90)	(0.24–21.86)
High-risk areas	1 [reference]	1 [reference]	1 [reference]	1 [reference]	1 [reference]	1 [reference]	1 [reference]	1 [reference]

OR, odds ratio; CI, confidence interval.

AOR, adjusted for gender, occupation, working department, years of service, ever contacted with COVID-19 patients and ever contacted with respiratory infectious diseases.

a Binary logistic regression model.

b Multinomial logistic regression model.

^c^In our study, occupation and year of service were matched.

#### Comparison of the items of depressive symptoms

Table 3 shows the results of the PHQ-9 scores of HCWs in matched samples. The total scores of PHQ-9 for HCWs working in HRAs and LRAs were 5.43 (SD 4.64), and 6.21 (SD 5.39), respectively. The PHQ-9 score of >10 was used as the cutoff value in this study to indicate a depressive symptoms tendency.

**Table 3:** Comparison of PHQ-9 scores of HCWs after matching^a^ (x ± SD).

Items	High-risk areas	Low-risk areas	*t*/*χ*^2^	*p* value
	(*n* = 146)	(*n* = 290)		
1. Little interest or pleasure in doing things	0.71 ± 0.73	0.89 ± 0.83	−2.235	0.026
2. Feeling down, depressed, or hopeless	0.60 ± 0.64	0.82 ± 0.78	−2.865	0.004
3. Trouble falling or staying asleep, or sleeping too much	1.10 ± 0.89	0.94 ± 0.91	1.684	0.093
4. Feeling tired or having little energy	0.87 ± 0.77	1.01 ± 0.92	−1.552	0.121
5. Poor appetite or overeating	0.73 ± 0.74	0.71 ± 0.81	0.283	0.777
6. Feeling bad about yourself -- or that you are a failure or have let yourself or your family down	0.33 ± 0.60	0.56 ± 0.72	−3.526	<0.001
7. Trouble concentrating on things, such as reading the newspaper or watching television	0.60 ± 0.83	0.63 ± 0.82	−0.340	0.734
8. Moving or speaking so slowly that other people could have noticed? Or the opposite --being so fidgety or restless that you have been moving around a lot more than usual	0.39 ± 0.59	0.46 ± 0.72	−1.001	0.318
9. Thoughts that you would be better off dead, or of hurting yourself in some way	0.10 ± 0.39	0.19 ± 0.51	−2.192	0.029
Total score	5.43 ± 4.64	6.21 ± 5.39	−1.557	0.120
Depressive symptoms	Number of HCWs (%)	Number of HCWs (%)		
Yes	22 (15.1%)	66 (22.8%)	3.703	0.054
No	124 (84.9%)	224 (77.2%)		

The items adapted from the PHQ-9 questionnaire (23, 24).^a^In our study, occupation and year of service were matched.

HCWs in LRAs had significantly higher scores than those in HRAs for Item 1, “little interest or pleasure in doing things” (0.89, SD 0.83 vs. 0.71, SD 0.73; *p* = 0.026), Item 2 “feeling down, depressed, or hopeless,” (0.82, SD 0.78 vs. 0.60, SD 0.64, *p* = 0.004), Item 6 “feeling bad about yourself or that you are a failure or have let yourself or your family down” (0.56, SD 0.72 vs. 0.33, SD 0.60, *p* < 0.001), and Item 9, “thoughts that you would be better off dead or of hurting yourself” (0.19, SD 0.51 vs. 0.10, SD 0.39, *p* = 0.029).

### Comparison of workplace environment characteristics

Table 4 shows the comparison of workplace environment characteristics of the HCWs in the matched sample. The N95 respirator was the most commonly used respiratory protection device in HRAs in both routine use (54%) and under specific conditions (55%; *p* < 0.001). Regarding personal protective equipment adequacy, a significant difference was found between HRAs and LRAs (76.0% vs. 52.1%, *χ*^2^ = 24.19, *p* < 0.001). Among HCWs, greater satisfaction with the provided infection control training was observed in those from HRAs (97.3%) rather than from LRAs (80.0%) areas (*χ*^2^ = 29.70, *p* < 0.001).

**Table 4:** Comparison of the HCWs’ workplace environment characteristics after matching^a^.

Variables	High-risk areas	Low-risk areas	*χ* ^2^	*p* value
	(*n* = 146)	(*n* = 290)		
Type of mask for routine use:			107.591	<0.001
General medical surgical mask	66 (45.2)	264 (91)		
N95 respirator	80 (54.8)	26 (9)		
Type of mask used in conditions (under specific conditions):			44.518	<0.001
General medical surgical mask	65 (44.5)	223 (76.9)		
N95 respirator	81 (55.5)	67 (23.1)		
Wished type of mask:			49.609	<0.001
General medical surgical mask	14 (9.6)	117 (40.3)		
N95 respirator	132 (90.4)	173 (59.7)		
Adequacy of personal protective equipment:			24.189	<0.001
Enough	111 (76)	151 (52.1)		
Not enough	35 (24)	139 (47.9)		
Satisfactory level of infection control training:			29.695	<0.001
Satisfactory	142 (97.3)	232 (80)		
Not satisfactory	4 (2.7)	58 (20)		

Variables adapted from a study by Bressington et al. (26).^a^In our study, occupation and year of service were matched.

### Comparison of scores on the health belief model

Table 5 presents the results of the HCWs’ scores in the HBM questionnaire. The following five dimensions of the HBM questionnaire showed significant differences between HCWs in HRAs and LRAs: perceived susceptibility (*t* = 2.253, *p* = 0.025), perceived severity (*t* = −4.895, *p* < 0.001), perceived barrier (*t* = 2.495, *p* = 0.013), cues to action for self-protection (*t* = 4.054, *p* < 0.001), and knowledge (*t* = 5.772, *p* < 0.001). Hence, the total scores also showed statistically significant differences (*t* = 3.493, *p* = 0.001). Apart from the dimension of perceived severity that the score from LRAs (4.93, SD = 1.63) was higher than that from HRAs (4.25, SD = 1.24), all the above mentioned dimensions obtained higher scores in HRAs than that in LRAs.

**Table 5:** Comparison of HCWs HBM scores after matching^a^ (x ± SD).

Dimensions	High-risk areas	Low-risk areas	*t*	*p* value
	(*n* = 146)	(*n* = 290)		
Perceived susceptibility	4.94 ± 0.94	4.72 ± 1.02	2.253	0.025
Perceived severity	4.25 ± 1.24	4.93 ± 1.63	−4.895	<0.001
Perceived benefits	3.57 ± 0.67	3.49 ± 0.65	1.186	0.236
Perceived barriers	5.76 ± 1.21	5.44 ± 1.36	2.495	0.013
Cues to action	7.29 ± 1.15	6.80 ± 1.33	4.054	<0.001
Knowledge	7.16 ± 1.00	6.49 ± 1.38	5.772	<0.001
Self-efficacy	3.77 ± 0.50	3.69 ± 0.51	1.45	0.148
Total score	36.73 ± 3.48	35.56 ± 3.21	3.493	0.001

Dimensions adapted from the health belief model (25).^a^In our study, occupation and year of service were matched.

### Subgroup analysis of associated factors to depressive symptoms

As the workplace environment characteristics of the HRAs and LRAs were heterogeneous, grouping all the samples for regression analysis is not recommended. Instead, subgroup analysis was used to delineate the corresponding associated factors in unmatched samples. Table 6 presents two individual results of binary logistic regression analyzes regarding factors related to depressive symptoms in HRAs and LRAs, respectively.

**Table 6:** Binary logistic regression examining factors related to depressive symptoms among high-risk areas (*n* = 146) and low-risk areas (*n* = 739).

Variables	High-risk areas			Low-risk areas		
	AOR	95%CI	*p* value	AOR	95%CI	*p* value
Basic characteristics						
Gender						
Male	Reference			Reference		
Female	0.27	0.06–1.32	0.106	0.79	0.45–1.39	0.415
Occupation						
Doctor	Reference			Reference		
Nurse	1.33	0.24–7.36	0.748	0.83	0.44–1.57	0.563
Other	0.39	0.01–30.86	0.670	0.52	0.22–1.24	0.142
Working department						
Internal medicine & surgery department	Reference			Reference		
Other	0.24	0.02–3.42	0.292	0.80	0.49–1.30	0.364
ICU	0.11	0.01–1.27	0.078	2.59	1.18–5.71	0.018
Pneumology department and Infectious Disease	0.06	0.00–0.95	0.046	0.47	0.17–1.32	0.151
Education level						
Associate degree or below	Reference			Reference		
Bachelor degree	0.57	0.05–6.37	0.644	0.80	0.49–1.32	0.387
Master degree or above	0.06	0.00–3.47	0.170	0.77	0.38–1.56	0.472
Marital status						
Unmarried/Other	Reference			Reference		
Married	3.65	0.54–24.49	0.183	0.92	0.54–1.59	0.777
Year of service						
<10	Reference			Reference		
10–20	6.27	1.07–36.70	0.042	1.10	0.64–1.89	0.740
>20	0.00	0.00-	0.998	1.11	0.57–2.19	0.757
Ever contacted with patients with COVID-19						
No	Reference			Reference		
Yes	14.33	1.20–171.62	0.036	1.35	0.83–2.18	0.223
Ever contacted with respiratory infectious diseases						
No	Reference			Reference		
Not sure	0.00	0.00-	0.999	1.06	0.43–2.63	0.897
Yes	0.11	0.00–2.62	0.171	1.14	0.62–2.09	0.678
Health Belief Model						
Perceived susceptibility	1.63	0.73–3.64	0.236	1.41	1.10–1.79	0.006
Perceived severity	0.93	0.46–1.87	0.829	1.25	1.07–1.46	0.006
Perceived benefits	0.40	0.12–1.30	0.126	1.04	0.73–1.48	0.846
Perceived barriers	4.48	1.82–11.05	0.001	1.43	1.21–1.69	<0.001
Cues to action	1.15	0.58–2.30	0.686	0.79	0.67–0.95	0.010
Knowledge	0.69	0.23–2.04	0.498	0.79	0.64–0.98	0.028
Self-efficacy	0.13	0.02–0.86	0.034	1.26	0.81–1.95	0.314

AOR, adjusted odds ratio.

For HCWs in HRAs, logistic regression showed that participants with years of service between 10 and 20 years (OR: 6.27, 95%CI = 1.07–36.70), ever had contacted with COVID-19 patients (OR: 14.33, 95%CI = 1.20–171.62), and had higher score of “perceived barrier” of HBM (OR: 4.48, 95%CI = 1.82–11.05) were at higher risk of depressive symptoms. Working in pneumology departments and infectious disease units (OR: 0.06, 95%CI = 0.00–0.95) and higher “self-efficacy” in the HBM (OR: 0.13 95%CI = 0.02–0.86) were protective factors against depressive symptoms.

For HCWs in LRAs, those who worked in ICUs (OR: 2.59, 95%CI = 1.18–5.72), had higher scores of “perceived susceptibility toward the COVID-19 outbreak” (OR: 1.41, 95%CI = 1.10–1.79), “perceived severity of the pandemic” (OR: 1.25, 95%CI = 1.07–1.46), and “perceived barriers of wearing masks” (OR: 1.43, 95%CI = 1.21–1.69) in the HBM were at higher risk of depressive symptoms. By contrast, HCWs who had higher scores of “cues to action for self-protection” (OR: 0.79, 95%CI = 0.67–0.95) and “knowledge of COVID-19” (OR: 0.79, 95%CI = 0.64–0.98) in the HBM were at lower risk of depressive symptoms.

## Discussion

This comparative study (HRAs vs. LRAs) adopted a cross-sectional design with matching essential socio-demographics in a 1:2 ratio, an approach which increases rigor in the method to reduce the risk of errors. According to the current result, a null hypothesis is hence rejected. Although a significant difference in depressive symptoms was found between HCWs working in HRAs and LRAs only in the unmatched sample, logistic regression analyzes indicated that the risk level of workplace significantly predicted depressive symptoms in both unmatched and matched samples. Surprisingly, HCWs in LRAs consistently expressed higher depressive symptoms than those in HRAs.

The impact of COVID-19 on HCWs’ psychological health (anxiety and depressive symptoms) has been reported in local (29, 30) and international studies (31–33). Studies have also reported high depressive symptoms (and high suicidal thoughts, measured by one item in the PHQ-9) among HCWs, particularly during the COVID-19 pandemic (31–33). To our knowledge, this research is one of the first to quantitatively compare the depressive symptoms among HCWs in HRAs and LRAs using a matched method. Utilizing these matched samples in a comparative study that minimized the selection bias of conveniently sampled HCWs would result in better credibility than a simple cross-sectional study, as the confounding factors have been adjusted (34, 35). After matching with occupation and years of service, the depressive symptoms among HCWs in LRAs were 1.96 times that of counterparts in HRAs.

### Overlooked group of healthcare workers

Depressive symptoms were common among HCWs during the initial stage of the pandemic (i.e., the first few months), irrespective of workplace environment characteristics. Studies conducted in other locations in Saudi Arabia, China, Europe, and Canada also found that HCWs had a high prevalence of depressive symptoms at 54, 43, 28, and 23%, respectively (36–39). Furthermore, in the United States, machine-learning analysis on the mental health of HCWs reported a decline in mental health associated mainly with the healthcare role of HCWs (i.e., Nurse, emergency room staff, etc.) (40). Various local research in China also established that HCWs were more likely to have depressive symptoms during the COVID-19 pandemic, a circumstance which was more common among women and nurses (29, 30, 41). The prevalence of depressive symptoms in this study was higher in LRAs than in HRAs (23.7% vs. 15.1%), an outcome which differs from the general perspectives and is new to the literature. HCWs in HRAs are believed to have a higher risk of depressive symptoms as they were facing more COVID-19 patients and witnessing much more death than their counterparts in LRAs, especially during the first month COVID-19 pandemic. However, our results revealed that HCWs working in LRAs during the initial stage of the COVID-19 pandemic had even higher depressive symptoms. Such a result might be related to insufficient attention or support.

### Justifications for the results

HCWs are at higher risk of depressive symptoms, particularly in the LRAs in our study. Several explanations may account for the current results.

First, we intentionally compared the workplace environment characteristics between HRAs and LRAs. We anticipated better quality, standard, and quantity of personal protective equipment and training in HRAs. Regarding the department/unit in which HCWs worked, most of the HCWs in LRAs worked in the internal medicine, surgery, and other departments. Additionally, HCWs exposed to COVID-19 patients were much fewer in LRAs than in HRAs (Table 1, 32.4% vs. 76.7%). Thus, we can infer that the samples of HCWs in HRAs in this study were indeed from HRAs. With reference to the principle of being “reasonably practicable” as resources for infection control and prevention (42), the current result is sound. Nevertheless, insufficient personal protective equipment is associated with depressive symptoms among HCWs regardless of the unit or workplace involved (20, 21, 42, 43). Therefore, clear instructions with justifications and the necessity of standard infection precaution and control are critical to strengthen the principle of being “reasonably practicable” and hence reduce unnecessary worry and anxiety.

Second, we also compared the scores of the HBM in our matched samples. The significant group difference for perceived severity indicated that HCWs in LRAs might overestimate the severity of COVID-19, thereby causing additional depressive symptoms. Coupled with their higher perceived barriers, fewer cues to action for self-protection, and less knowledge on COVID-19, HCWs in LRAs demonstrated more depressive symptoms. All of these associated factors were salient predictors in the logistic regression model.

Lastly, the univariate analysis indicated that the difference in depressive symptoms was marginally not significant between matched HCWs in HRAs and LRAs (*p* = 0.054). However, after adjusting for several essential confounders (i.e., gender, occupation, working department, years of service, ever contacted with COVID-19 patients and ever contacted with respiratory infectious diseases), both unmatched and matched samples demonstrated a consistent and significant result that HCWs in LRAs had a higher risk of depressive symptoms than those in HRAs. This inconsistency demonstrated the limitation of the univariate analysis and the strength of the multivariate analysis.

In HRAs, this result is consistent with those reported by Luo and his teammates (29). HCWs in HRAs were under immense pressure to work with many COVID-19 patients and diagnose and treat highly infectious COVID-19 cases. Therefore, depressive symptoms were intuitively understandable, as reported by numerous studies (30, 39–41, 44). In contrast to their high-risk counterparts, HCWs in LRAs were less likely to be exposed to COVID-19 patients. However, diversity and workload intensity have been added as variables because of shift scheduling from those HCWs assigned to HRAs. According to the literature, depressive symptoms among HCWs might be attributed to longer working hours, responsibility for and contact with more patients, insufficient personal protective equipment, inadequate infectious control training, and frequently witnessing the death of patients (7–9, 17, 45, 46). Some associated factors, like working on the frontline and worried about infection, were consistently indicated by a multidimensional machine learning-based prediction model (28).

### Recommendations

HCWs in HRAs received widespread public attention and emotional support, such as national awards and commendations, gratitude, salary improvement, and welfare (47, 48). By contrast, HCWs in LRAs did not receive adequate public attention and emotional support, which might explain why they were being overlooked in relation to their contribution as well as their needs.

Thus, governments, health administrations, and society should also pay appropriate attention to HCWs in LRAs as they do for HCWs in HRAs. Hospital leaders should enhance support in the workplace, including improving infection control of COVID-19 and psychological training for HCWs. Furthermore, strategies should be implemented to reduce the working hours for one shift, increase subsidy, and provide HCWs preferential treatment in professional title appraisals. Hospital leaders should also support HCWs with a sense of professionalism, mission, and honor to keep improving their psychological health.

### Limitations

This study had several limitations. First, the HCWs in the LRAs only came from the Guangdong–Hong Kong–Macao Greater Bay Area. This geographical limitation affects the representativeness of the HCWs. Second, the HCWs in LRAs were mostly from other and internal medicine departments compared to HCWs in HRAs who were mostly from intensive care units and infectious disease units, a discrepancy which might lead to the underestimation of the prevalence of depressive symptoms in the LRAs group compared to their high-risk counterparts. However, our study revealed a higher tendency of depressive symptoms among HCWs in LRAs relative to those from HRAs, an outcome which may somewhat attenuate the working department difference of HCWs from the two areas. Moreover, this underestimation of the prevalence of depressive symptoms in LRAs would generate greater attention toward the psychological health prevention strategy of HCWs in LRAs during the COVID-19 pandemic. Finally, given the self-report questionnaires and online-based approach applied for data collection in this study, recall and selection biases are possible. Although we applied a matched sampling method and adjusted for confounding factors during analysis, those measures cannot completely rectify these biases.

## Conclusion

The prevalence of depressive symptoms among HCWs in LRAs was significantly higher than that in HRAs. Associated factors of depressive symptoms among HCWs in LRAs and HRAs were different. Hence, subgroup analyzes were recommended to delineate their salient predictors. In general, results suggested a need for a holistic approach, such as providing adequate personal protective equipment and suitable infection control training and support, to reduce the risk of depressive symptoms among HCWs whether they are employed in HRAs or LRAs.

## Data availability statement

The original contributions presented in the study are included in the article/Supplementary material, further inquiries can be directed to the corresponding authors.

## Ethics statement

The studies involving human participants were reviewed and approved by Human Subjects Ethics Sub-committee of the Special Geriatric Committee of Zhongshan Medical Association. The patients/participants provided their written informed consent to participate in this study.

## Author contributions

EH, SL, and LS were responsible for the conceptualization and study design. EH, SC, VP, and JH collected the data. EH, LL, and X-WZ handled the preparation of the first draft of the manuscript. X-WZ analyzed the data. SL and LS provided a critical review to finalize the draft. All authors provided intellectual content, reviewed the manuscript, and agreed on the submitted version.

## Funding

This study received funding from the Research Foundation for Talented Scholars of Zhongshan Polytechnic (Grant no. KYG2107). The funding sources were not involved in the study design; data collection, analysis, and interpretation; manuscript drafting; or submission decisions.

## Conflict of interest

The authors declare that this research was conducted without any commercial or financial relationships that could be construed as potential conflicts of interest.

## Publisher’s note

All claims expressed in this article are solely those of the authors and do not necessarily represent those of their affiliated organizations, or those of the publisher, the editors and the reviewers. Any product that may be evaluated in this article, or claim that may be made by its manufacturer, is not guaranteed or endorsed by the publisher.

## Abbreviations

HCWs: Healthcare Workers
HRAs: High-risk areas
LRAs: Low-risk areas
COVID-19: Coronavirus disease
PHQ-9: Patient Health Questionnaire-9
HBM: Health Belief Model
