# Supplementary material for: Comparison of depressive symptoms among healthcare workers in high-risk versus low-risk areas during the first month of the COVID-19 pandemic in China
Source: Front Psychiatry. 2023 Jun 13;14:1154930. doi: 10.3389/fpsyt.2023.1154930 (PMC10293622; doi:10.3389/fpsyt.2023.1154930)
Supplement: Supplementary file 1 [file Table_1.DOCX]

**Supplementary Table A.** Study places of the HCWs

| Study places (number of HCWs) | Low-risk area* | High-risk area* |
| --- | --- | --- |
| Hebei Province | 9 | 0 |
| Guangdong–Hong Kong–Macao Greater–Bay–Area |  |  |
| - Hongkong | 131 | 1 |
| - Macau | 23 | 0 |
| - Guangdong Province | 556 | 4 |
| Hunan Province | 3 | 0 |
| Hubei Province | 17 | 141 |
| Total: | 739 | 146 |

*Remarks: The definition and characteristics can be referred to Supplementary Table B

**Supplementary Table B** Description of grouping criterion of the high-risk areas and low-risk areas in this study

| COVID-19 outbreak in his/her working regions | Working place where the HCW worked with the COVID-19 patients | Eligible | Grouping | Example |
| --- | --- | --- | --- | --- |
| No | No | Yes | Low-risk areas | There is no COVID-19 outbreak in Tibet Autonomous Region in March 2020. Thus, any HCWs recruited in this region will be grouped under low-risk areas. |
| No | Yes | Impossible | NA | There is no COVID-19 outbreak in Tibet Autonomous Region in March 2020. Thus, it is impossible to have a HCWs taking care of a COVID-19 patient in this region. |
| Yes | No | Yes | Low-risk areas | There is a recognized COVID-19 outbreak in Hong Kong and a HCW is taking care of his/her patients in the rehabilitative ward where no COVID-19 case was found in there. |
| Yes | Yes | Yes | High-risk areas | There is a recognized COVID-19 outbreak in Hong Kong and a HCW is taking care of his/her COVID-19 patients in the intensive care unit. |
