# Supplementary material for: Comparison of depressive symptoms among healthcare workers in high-risk versus low-risk areas during the first month of the COVID-19 pandemic in China
Source: Front Psychiatry. 2023 Jun 13;14:1154930. doi: 10.3389/fpsyt.2023.1154930 (PMC10293622; doi:10.3389/fpsyt.2023.1154930)
Supplement: Supplementary file 2 [file Data_Sheet_1.docx]

Supplementary materials C: Illustration of demographics in high-risk and low-risk areas

**Gender**

**Occupation**

**Working department**

**Education level**

**Marital status**

**Year of service**

**Contact with COVID-19 patients**

**Contact with respiratory infectious diseases**

**Depressive symptoms**

**Severity of depressive symptoms**
